# Genomic distribution of AFLP markers relative to gene locations for different eukaryotic species

**DOI:** 10.1186/1471-2164-14-528

**Published:** 2013-08-01

**Authors:** Armando Caballero, María Jesús García-Pereira, Humberto Quesada

**Affiliations:** 1 Departamento de Bioquímica, Genética e Inmunología, Facultad de Biología, Universidade de Vigo, 36310, Vigo, Spain

**Keywords:** AFLP, Candidate genes, Genome scans, Genomic signature, Restriction-site markers

## Abstract

**Background:**

Amplified fragment length polymorphism (AFLP) markers are frequently used for a wide range of studies, such as genome-wide mapping, population genetic diversity estimation, hybridization and introgression studies, phylogenetic analyses, and detection of signatures of selection. An important issue to be addressed for some of these fields is the distribution of the markers across the genome, particularly in relation to gene sequences.

**Results:**

Using *in-silico* restriction fragment analysis of the genomes of nine eukaryotic species we characterise the distribution of AFLP fragments across the genome and, particularly, in relation to gene locations. First, we identify the physical position of markers across the chromosomes of all species. An observed accumulation of fragments around (peri) centromeric regions in some species is produced by repeated sequences, and this accumulation disappears when AFLP bands rather than fragments are considered. Second, we calculate the percentage of AFLP markers positioned within gene sequences. For the typical *Eco*RI/*Mse*I enzyme pair, this ranges between 28 and 87% and is usually larger than that expected by chance because of the higher GC content of gene sequences relative to intergenic ones. In agreement with this, the use of enzyme pairs with GC-rich restriction sites substantially increases the above percentages. For example, using the enzyme system *Sac*I/*Hpa*II, 86% of AFLP markers are located within gene sequences in *A. thaliana*, and 100% of markers in *Plasmodium falciparun*. We further find that for a typical trait controlled by 50 genes of average size, if 1000 AFLPs are used in a study, the number of those within 1 kb distance from any of the genes would be only about 1–2, and only about 50% of the genes would have markers within that distance.

**Conclusions:**

The high coverage of AFLP markers across the genomes and the high proportion of markers within or close to gene sequences make them suitable for genome scans and detecting large islands of differentiation in the genome. However, for specific traits, the percentage of AFLP markers close to genes can be rather small. Therefore, genome scans directed towards the search of markers closely linked to selected loci can be a difficult task in many instances.

## Background

Amplified fragment length polymorphisms (AFLP; [1]) are extensively used in evolutionary, population genetics and conservation studies on plants, animals and microorganisms [2,3]. Applications of these markers are particularly useful in non-model species for which no prior DNA sequence is available, and where other alternative wide-genome markers, such as SNPs, are difficult to obtain. AFLP markers are also very useful because of their low cost relative to other markers [4]. Thus, AFLP markers have been used for a wide range of objectives, such as genome-wide mapping (e.g. [5]), population genetic diversity estimation, hybridization and introgression studies (e.g. [6-8]), phylogenetic analyses (e.g. [9-11]) and detection of signatures of selection (e.g. [12-17]). More recently, restriction site associated DNA markers (RAD; [18,19]) have been suggested as an alternative tool for some of the above objectives, although important problems also affect this type of marker [20,21].

Several concerns regarding the application of AFLP markers have been addressed and discussed in the recent years. One is the possible lack of homology due to fragment size homoplasy [16,22-25]. Homoplasy may produce biases in the estimates of population genetic parameters [22,26], in the efficiency of the methods to detect loci under positive selection in genome-wide scans [26], and in phylogenetic reconstruction (e.g. [9,27-30]). However, the use of homoplasy-corrected estimators of genetic similarity from AFLP bands [31] and the use of a restricted number of markers per primer combination [1,2,28] allows for a minimization of the impact of homoplasy on the multiple applications of AFLP markers. Other concerns regarding AFLP markers are the difficulties in isolating and characterising AFLP loci [32] and the possible problems due to insufficient fragment mobility resolution or an incorrect scoring of bands [33]. Some of these problems are currently addressed by new scoring method proposals [34-36] or quantitative genetic approaches [7].

A further issue to be addressed in the use of AFLP markers, particularly regarding their applications in QTL mapping and detection of signatures of selection, is the distribution of the markers across the genome. Although AFLP markers are assumed to offer a good genomic coverage, it has been reported that they are frequently clustered around centromeric regions (e.g. [37-39]). In addition, several studies recognize the presence of over- and under-representation of short oligonucleotides in DNA sequences that can be regarded as a genomic signature of the species (e.g. [40-42]) and could affect the distribution of AFLP markers across the genome. In fact, neither the distribution of AFLP fragment lengths nor the distribution of AFLP positions across the genome are random [23,24]. Finally, it has been repeatedly seen that gene concentration increases from GC-poor to GC-rich regions of the eukaryotic genomes (e.g. [43,44]). Thus the ability of restriction-site markers to be localised in gene or intergene sequences should depend on the restriction enzymes used.

In QTL mapping studies as well as in analyses of detection of loci under selection in genome-wide scans, hundreds or thousands of markers are used with the aim of finding markers associated to the loci of interest. The association is made through the observation of a correlation between markers and the trait of interest in the first case, or the observation of a high level of differentiation among populations for the markers in the second. Many of these studies are carried out with restriction site markers, particularly AFLPs, and it is relevant to know whether the distribution of these markers is suitable for such studies. For example, recent extensive genome scans indicate that genetic differentiation of markers attached to selected regions does not extend beyond about 1–5 kb around the adaptive loci [45]. It is thus important to have *a priori* predictions of the upper number of markers expected to be within or close to the genes of interest.

In this paper we focus on the above issues analysing whole genome sequences and data on gene positions on the genome from different eukaryotic species. We first identify the physical position of AFLP fragments across the chromosomes of nine sequenced eukaryotic species to check their genome coverage. Second we compute the physical distance between AFLP markers and their nearest genes in order to see the proportion of markers physically associated to genes. Finally, we illustrate the relative position of AFLP markers with respect to specific sets of genes controlling a particular trait of interest.

## Results

### Distribution of AFLP markers across the genome

**Figure 1:**
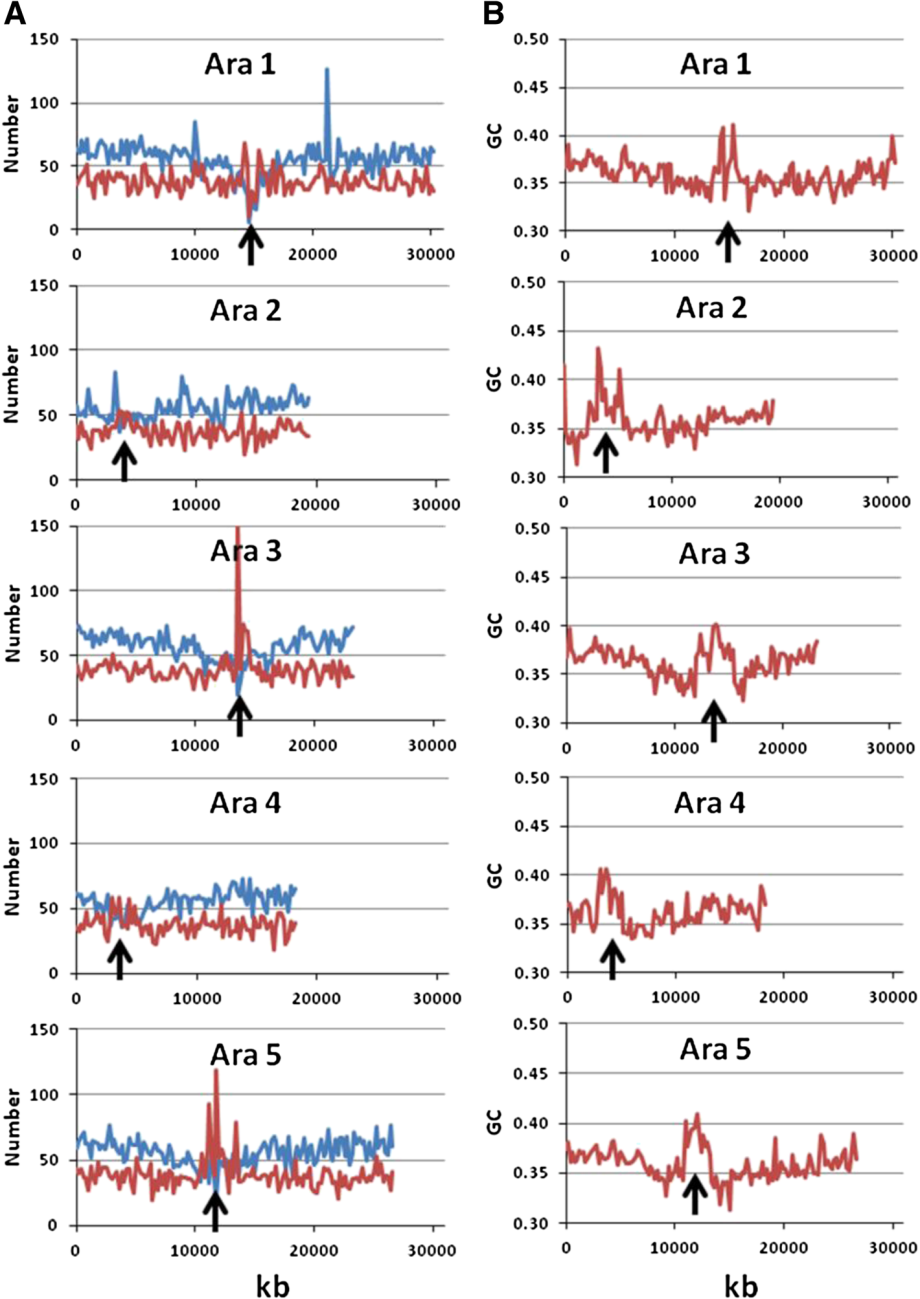
**Distribution of the number of AFLP fragments, number of genes, and average GC content, across the different chromosomes of *Arabidopsis thaliana*, shown in non-overlapping windows of 200 kb. (A)** Number of AFLP fragments (EcoRI/MseI) in red, number of genes in blue. **(B)** Average GC content. The approximate location of the centromeric regions is marked with an arrow.

**Figure 2:**
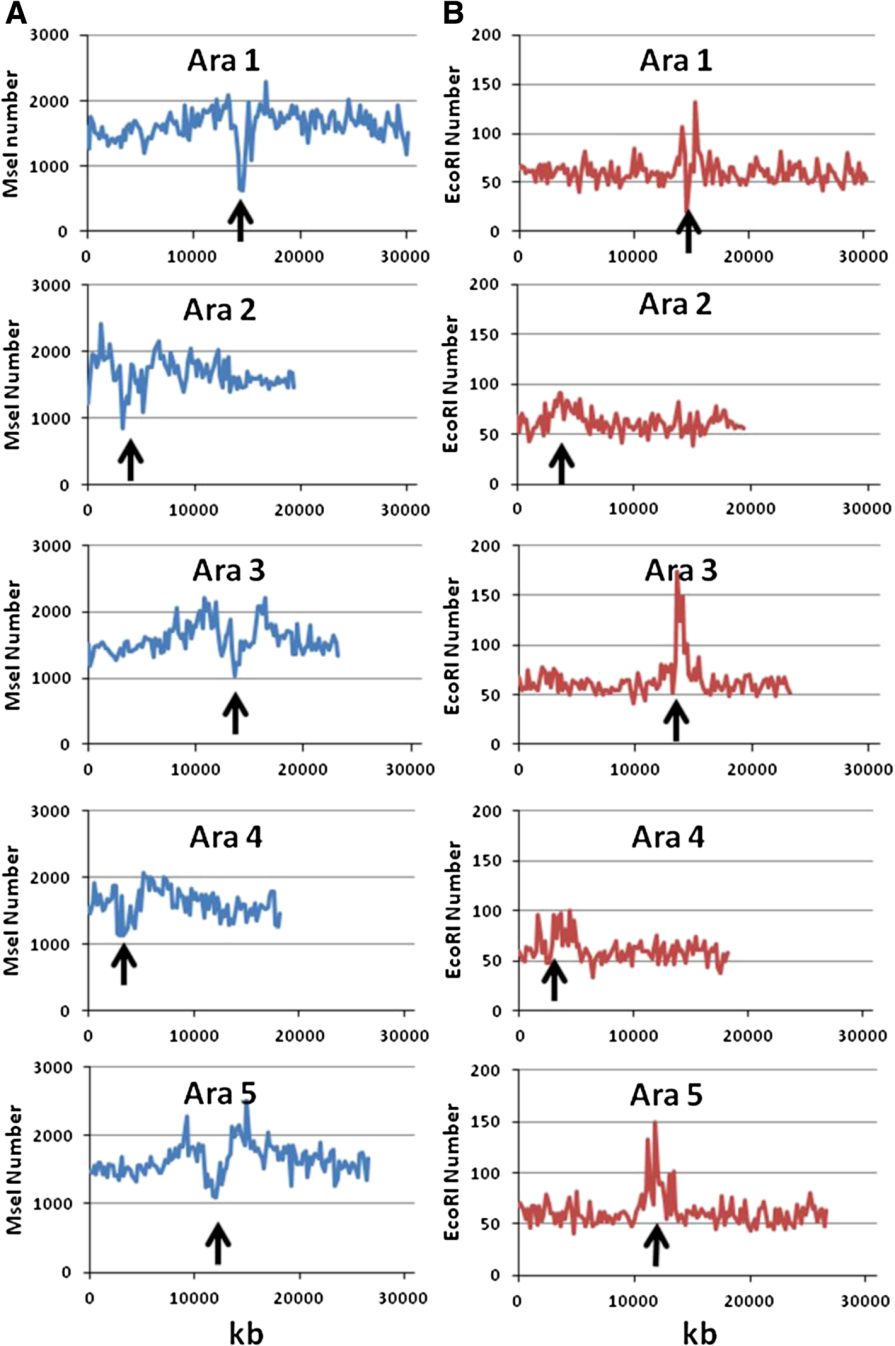
**Distribution of the number of *Mse*I (A) and *Eco*RI (B) cutting sites across the different chromosomes of *Arabidopsis thaliana*, shown in non-overlapping windows of 200 kb.** The approximate location of the centromeric regions is marked with an arrow.

**Figure 3:**
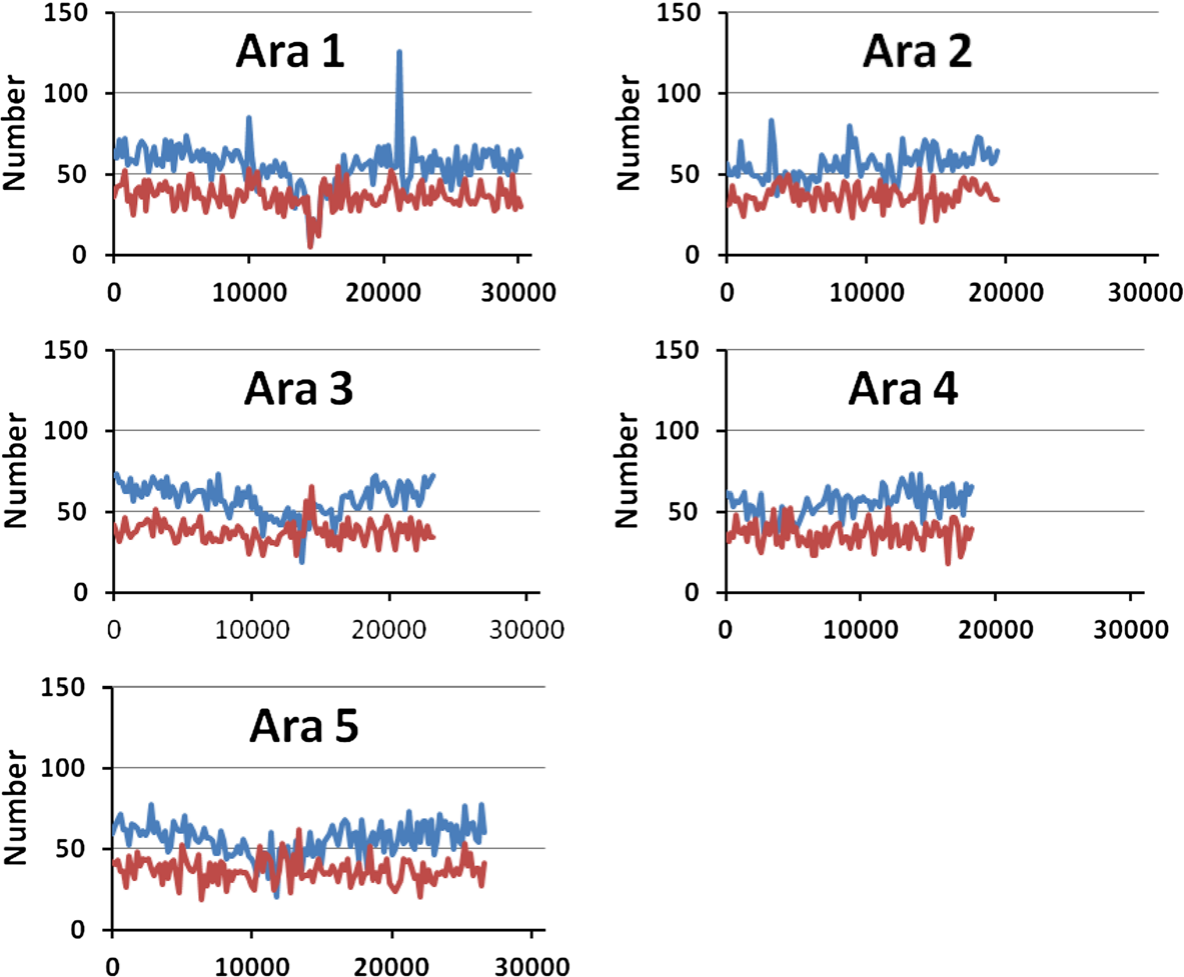
**Distribution of the number of AFLP bands (*Eco*RI/*Mse*I) (in red) and the number of genes (in blue) across the different chromosomes of *Arabidopsis thaliana*, shown in non-overlapping windows of 200 kb.**

We first focus on the *Arabidopsis thaliana* genome, as a number of *in-silico* studies have been carried out previously on this species. The distribution of the number of AFLP fragments (*Eco*RI/*Mse*I) and the number of genes across the different chromosomes are shown in non-overlapping windows of 200 kb in Figure 1A. It is apparent that a certain accumulation of AFLP fragments are located around or in the centromeric regions, particularly for chromosomes 3 and 5. The reason for these increases in the number of fragments can be ascribed to the higher GC content attached to these genomic areas (Figure 1B). Indeed, although the number of *Mse*I sites is lower in these regions than in others (Figure 2A), the number of *Eco*RI sites they contain is drastically increased (Figure 2B), leading to an increase in the number of AFLP fragments. Nevertheless, the excess of AFLP fragments around the centromeric regions, virtually disappears when AFLP bands rather than fragments are considered in the analysis (Figure 3). The reason is that in the centromeric regions repeated sequences which produce particular fragments of the same size occur and can be expected to collide in the same electrophoretic band. In order to check this explanation, we looked in detail at the centromeric regions of chromosomes 3 and 5 as defined by The Arabidopsis Genome Initiative [46]. We found, for example, that an AFLP fragment sequence of 104 bp in the centromeric region of chromosome 3 repeated 50 times. In chromosome 5 there was an AFLP fragment sequence of 117 bp repeated 63 times and one of 116 bp repeated 9 times.

The distribution of AFLP bands and genes for the other analyzed species are given in the Additional file 1: Figures S1-S8. In general, no regions with extreme accumulation of AFLP bands were observed.

### Distance between AFLP markers and genes for the whole genome

The first row of Table 1 shows the total genome length available and analyzed for each of the species. The percentage of un-sequenced nucleotides was relatively small in all cases (7.79% in *Homo*, 2.63% in *Oryza*, 2.36% in *Anopheles*, 0.08% in *Drosophila*, 0.16% in *Arabidopsis*, 0% in *Caenorhabditis*, 0.004% in *Plasmodium*, 0.003% in *Schizosaccharomyces*, and 0% in *Saccharomyces*). The results presented below are not affected by these un-sequenced nucleotides because AFLPs, gene locations and their distances obviously refer only to sequenced areas, with un-sequenced nucleotides generally being clustered in large regions. The second and third rows show the GC content for each species for gene and intergene sequences. Note that the GC% is consistently larger for the former than for the latter. The next two rows show the total number of genes and the gene length mean and its standard deviation.

**Table 1:** ***In-silico *analysis of whole genome sequences from 9 eukaryotic species ( *Homo sapiens, Oryza sativa *, *Anopheles gambiae*, *Drosophila melanogaster*, *Arabidopsis thaliana*, *Caenorhabditis elegans*, *Plasmodium falciparum*, *Schizosaccharomyces pombe *and *Saccharomyces cerevisiae*)**

	**Homo**	**Oryza**	**Anoph.**	**Droso.**	**Arab.**	**Caeno.**	**Plasm.**	**Schizo.**	**Sacch.**
Genome size (Mb)	3003	382	230	120	119	100	23	13	12
GC% (gene sequences)	0.419	0.446	0.473	0.437	0.393	0.364	0.227	0.388	0.396
GC% (intergenic sequences)	0.402	0.432	0.433	0.403	0.313	0.341	0.144	0.345	0.347
Number of genes	36036	30295	12688	14604	33239	21175	5509	5060	6281
Mean (stand. dev.) gene length (kb)	35.5 (81.2)	3.0 (2.6)	5.9 (4.5)	6.3 (4.7)	2.1 (1.6)	2.9 (3.3)	2.5 (2.6)	1.4 (0.7)	1.4 (1.2)
**Enzymes *Eco*RI/ *Mse *I**									
Number of AFLPs	459944	50437	28336	20767	22836	27345	2017	2748	2891
Mean distance between AFLPs (kb)	6.7	7.4	8.0	5.7	5.1	3.5	11.2	4.4	4.1
**% AFLPs at a given distance from genes**									
0 kb (EXP*)	41 (*43*)	29 (*25*)	28 (*32*)	63 (*63*)	67 (*59*)	65 (59)	79 (*59*)	71 (*60*)	87 (*73*)
1 kb	43	42	36	71	89	83	96	94	99
10 kb	53	80	64	92	99	100	100	99	100
**% Genes with AFLPs at a given distance**									
0 kb (EXP**)	48 (*99*)	33 (*38*)	27 (*46*)	35 (*59*)	34 (*37*)	47 (57)	23 (*25*)	31 (*32*)	31 (*33*)
1 kb	63	50	45	56	55	70	31	56	57
10 kb	92	95	93	96	98	100	85	100	99
**Enzymes *Bsm*I/ *Taq *I**									
Number of AFLPs	101630	31357	35330	21155	10441	12518	464	1751	1475
% AFLPs at 0 kb from genes	45	30	33	64	73	69	92	76	84
**Enzymes *Sac*I/ *Hpa *II**									
Number of AFLPs	131756	45330	17529	10234	6579	7098	19	406	467
% AFLPs at 0 kb from genes	52	29	39	72	86	75	100	89	87

* Expected value calculated as (*Mean gene length* × *Number of genes*)/*sequenced genome size*.

** Expected value calculated as 1 – exp[−*average number of AFLPs within genes*].

The next block of rows shows results for AFLP fragments cut by enzymes *Eco*RI/*Mse*I. Note that the total number of AFLP fragments is generally larger than the number of genes for species with large genome sizes, but the mean distance between AFLPs is relatively uniform across all species, with most values ranging between about 4 and 8 kb.

Next, the table presents the percentage of AFLP fragments positioned at a given physical distance from the closest gene. AFLP markers at a 0 kb distance from genes refer to those within the gene sequence or over-lapping it. The expected value of this percentage if AFLP fragments were randomly positioned in the genome is shown in parenthesis. This expectation is simply calculated as the percentage of the sequenced genome covered by all gene sequences. For 6 out of 9 species the observed percentage is larger than the random expectation. AFLP markers at 1 kb distance from genes include also those at 0 kb distance, etc.

**Figure 4:**
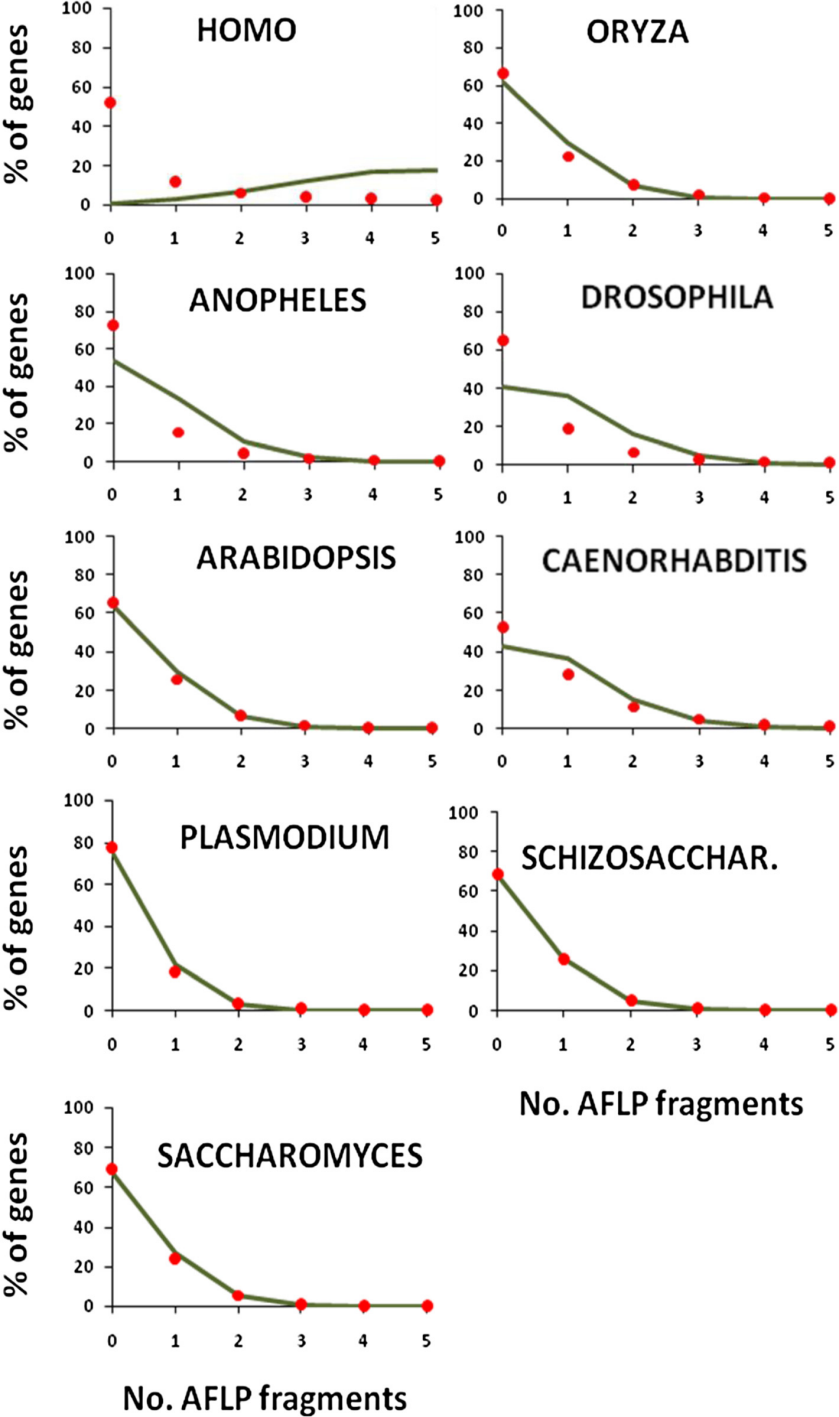
**Distribution of the observed percentage of genes (red dots) with a given number of AFLP fragments (*Eco*RI/*Mse*I) within their sequence.** The line gives the expectation under a Poisson distribution.

The next group of rows in Table 1 shows the percentage of genes with AFLP fragments at a given distance. The percentage of genes with AFLPs at 0 kb distance indicates those genes with at least one AFLP fragment inside the gene sequence. The expectation of this value, given in parenthesis, is the Poisson expectation with the observed mean number of AFLP fragments per locus. For all species the observed percentage is lower than the expected value. The percentage of genes with zero, one, two, etc. AFLP fragments inside gene sequences is given in Figure 4. The discrepancy between observed and expected values can be ascribed to the fact that the poisson expectation assumes equal gene length sequence for all genes, a clearly untrue assumption, particularly for the human genome. Note that the percentage of genes having AFLP fragments below 1 kb distance is around 50-60% for most of the species (Table 1).

All the above results refer to AFLP fragments using the typical tandem *Eco*RI/*Mse*I. The four last rows of Table 1 show some results for tandems with a balanced AT/GC (*Bsm*I/*Taq*I) or a GC biased (*Sac*I/*Hpa*II) recognition sequence. The number of AFLP fragments is normally decreased (although, for some species, increased) with the GC content of the restriction sites (2/10 GC nucleotides for *Eco*RI/*Mse*I, 5/10 for *Bsm*I/*Taq*I, and 8/10 GC for *Sac*I/*Hpa*II). Note that the percentage of AFLP fragments inside gene sequences is increased with an increase of the GC content of the restriction sites for all cases except for *Oryza*. In addition, the use of selective G/C nucleotides slightly increases this percentage. For example, using the pair *Eco*RI/*Mse*I with one selective nucleotide (G or C) at each extreme of the fragment, the percentage of AFLP fragments inside gene sequences increases from 27% (no selective nucleotides) to 29% (G or C selective nucleotides) for *Anopheles*, and from 65% to 66% in *Caenorhabditis*. Using the pair *Sac*I/*Hpa*II the corresponding increases were from 39% to 46%, and from 75% to 77%, respectively.

### Examples of distances between AFLP markers and genes for specific traits

In order to illustrate the availability of AFLP markers close to a specific set of genes, we considered three examples of candidate genes in three of the species analysed above (Table 2). The distribution among chromosomes of 42 candidate genes for Aluminium tolerance in *Oryza sativa* is 7, 5, 5, 3, 4, 2, 3, 0, 2, 5, 3 and 3 for chromosomes 1 to 12, respectively; that of 50 candidate genes for flowering time in *Arabidopsis thaliana* is 9, 9, 7, 12 and 13 for chromosomes 1 to 5, respectively; and that for 89 candidate genes for developmental time in *Drosophila melanogaster* is 12, 16, 21, 15 and 25 for chromosomes 2L, 2R, 3L, 3R and X, respectively.

**Table 2:** ***In-silico *analysis of candidate genes for Aluminium tolerance (AL) in *Oryza sativa*, developmental time (DT) in *Drosophila melanogaster*, and flowering time (FT) in *Arabidopsis thaliana***

	**Oryza (AL)**	**Droso (DT)**	**Arab (FT)**
Number of candidate genes	42	89	50
Mean (stand. dev.) gene length (kb)	3.4 (2.0)	30.4 (30.2)	3.4 (1.8)
**% AFLPs at a given distance from genes**			
0 kb (EXP*)	0.06 (*0.04*)	1.89 (*2.05*)	0.14 (*0.14*)
1 kb	0.09	2.02	0.24
10 kb	0.30	3.32	0.95
100 kb	2.39	14.73	7.51
**% Genes with AFLPs at a given distance**			
0 kb (EXP**)	43 (*50*)	73 (*99*)	34 (*47*)
1 kb	57	80	55
10 kb	86	99	98
100 kb	100	100	100

* Expected value calculated as (*Mean gene length* × *Number of candidate genes*)/*sequenced genome size*.

** Expected value calculated as 1 – exp[−*average number of AFLPs within candidate genes*].

The average gene length of the *Drosophila* candidate genes for developmental time is particularly large (30.4 kb; about 5 times larger than the average gene length for the species; Table 1) implying that about 2% of AFLP fragments could be located within 1 kb of the candidate genes, and 80% of the candidate genes would have possible markers within a 1 kb distance. However, these figures are substantially lower for the other examples, which give gene lengths of more average size (about 3.4 kb; somewhat above the mean gene lengths for the species; see Table 1). Thus, only 1 or 2 AFLP fragments out of 1000 would be expected to be within a 1 kb distance from any of the candidate genes in the Aluminium tolerance or flowering time examples in *Oryza* and *Arabidopsis*, respectively; and only about 50% of the candidate genes would have possible markers at a 1 kb distance from them.

## Discussion

AFLP markers are considered to be widely distributed across the genome [3] and thus to be useful markers for genome-wide scan studies for a variety of objectives, such as gene mapping, detection of signatures of selection and hybridization and introgression. However, it is well-known that the genomic sequences of many organisms display internal heterogeneities of different kinds, including variation in GC content, coding versus non coding sequences, hierarchies of repeats, etc. [47]. In fact, the distribution of AFLP fragments significantly deviates from that expected at random (e.g. [48-51]). Using *in-silico* analyses of different species it has been shown that the internal compositional heterogeneity of the genomes is responsible for the non-random physical distribution of AFLP markers [23].

The observation that many AFLP markers cluster around centromeric regions in genetic maps, as reported in *Arabidopsis*[37,39], potato, [48], soybean [50,51], wild emmer wheat [38], pink salmon [49], etc. is of particular interest. However, because this clustering has been observed in genetic maps, it was not possible to ascribe it only to a reduced recombination rate in these regions (e.g. [50,51]) or to a higher frequency of markers. In an important study addressing this issue, Peters et al. [39] carried out a combination of *in-silico* restriction fragment analysis and experimental AFLP analysis in *Arabidopsis thaliana* using *Sac*I/*Mse*I enzymes. They were able to find the physical position of 1267 experimental AFLP markers in the genome, showing that 98.6% of the genome is covered by AFLPs. They showed that a reduced recombination rate in (peri) centromeric regions was only part of the explanation for the observed accumulation of AFLPs in these regions. In physical maps, there was still some agglomeration of empirical AFLP markers around centromeric regions. Nevertheless, Peters et al. [39] indicated that the occurrence of *in-silico* AFLP fragments was not increased in the (peri) centromeric regions, although this observation was not explicitly shown in the article. Here we have revisited the point regarding the typical enzyme system *Eco*RI/*Mse*I and found an increase in the number of AFLP markers in the (peri) centromeric regions of some chromosomes, particularly chromosomes 3 and 5 (Figure 1A). This was shown to be both a consequence of the higher GC content in these regions (Figure 1B and 2) and the presence of some repeated sequences which generate the same fragments. When AFLP bands rather than fragments are considered, which is more appropriate for an experimental setting, the (peri) centromeric agglomerations of AFLP markers mostly disappear (Figure 3). Thus, AFLP markers do not particularly accumulate in some regions of the genome. However, in experimental analyses, they still appear somewhat more frequently in the (peri) centromeric regions. Peters et al. [39] suggested that the explanation for this empirical observation may be that the frequency of mutations is increased in these regions. This is in fact a highly reasonable explanation, as it may be expected that the degree of polymorphism is larger in (peri) centromeric regions than in other coding sequences, so that segregating AFLP markers are more likely to be found in the former. In summary, the observed accumulation of empirical AFLP markers in (peri) centromeric regions can be due to a reduced recombination rate (for genetic maps; e.g. [50,51]) and a higher polymorphism (for genetic and physical maps [39]) in these regions. However, the physical distribution of AFLP markers, although non-random (e.g. [23,24]) has a coverage wide enough so as to become useful markers in genome-scan studies.

Regarding the location of AFLP markers relative to gene positions, we have shown that for the *Eco*RI/*Mse*I system the percentage of AFLP markers located within gene sequences ranges between 28% and 87% depending on the species and it is somewhat larger than expected by chance. The reason is likely to be that the GC content for gene sequences is generally larger than for intergene sequences (e.g. [43,44,52]), and this increases the likelihood of enzyme cuts in the former. The use of enzymes with a higher GC content (*Bse*I/*Taq*I and *Sac*I/*Hpa*II) further increases this likelihood. It is remarkable that, for example, using the pair *Sac*I/*Hpa*II in *Arabidopsis*, 86% of the 6579 possible AFLP fragments are located within gene sequences, rising to 95% for fragments located within 1 kb distance from genes. These results are in agreement with those of Arnold et al. [21] in their analysis of the biases associated with RAD markers for the estimation of diversity. In their study, *in silico* digestion of *D. melanogaster* genomes indicated that GC-rich recognition sequences appear more frequently in exons, whereas AT-rich recognition sequences appear disproportionately more in intronic and intergenic regions. Therefore, we can conclude that using enzymes with high GC content could be more appropriate than enzymes with low GC content if the objective is to get available markers as close as possible to gene sequences.

The number of AFLP fragments clearly depends on the genome size, showing a rather linear relationship. The regression of the number of AFLP markers (*Eco*RI/*Mse*I) on genome size for the nine species analysed has a slope of 152 markers per megabase with a squared correlation of *R*^2^ = 0.998. If the human genome is excluded in the analysis, the slope is a bit lower, 125 markers per megabase, with *R*^2^ = 0.900. Thus, the density of AFLP markers is of about one AFLP per 7 kb. Using the enzymes *Bsm*I/*Taq*I and *Sac*I/*Hpa*II, the corresponding slopes (including all 9 species) are 31 (*R*^2^ = 0.908) and 43 (*R*^2^ = 0.953) markers per megabase, respectively, implying densities of about one AFLP per 32 kb for *Bsm*I/*Taq*I and about one AFLP per 23 kb for *Sac*I/*Hpa*II. The corresponding densities in the genetic map vary substantially among species. For example, in *Oryza* and *Arabidopsis* 1 cM corresponds to about 200–250 kb on average [39,53]. Thus, with *Eco*RI/*Mse*I it is expected to be about 30 AFLPs per centimorgan for these species. However, in *Drosophila* 1 cM corresponds to about 0.63 Mb of sequence on average, and in Humans 0.82 Mb [54]. Thus, in these cases, there is an expected number of about 100 AFLPs per centimorgan. In general, therefore, the density of AFLP markers is relatively high, making AFLP markers generally suitable for genome scans.

When specific traits are considered, however, the percentage of AFLP markers within gene sequences or close to them can be rather small. We have illustrated this with some examples in three of the species analysed (Table 2). The results show that, for a typical trait controlled by a few dozen of genes of the typical gene size in the species, the number of AFLPs within 1 kb distance from those genes can be of the order of 1–2 in an AFLP analysis involving 1000 markers. In addition, only about 50% of the genes of interest would have markers within that distance. Thus, genome scans directed towards the search of markers closely associated to specific selected loci can be difficult depending on the situations. For example, genomic scans using molecular markers, such as AFLPs, are frequently used to infer adaptive population divergence [55-57]. Some of the methods used are based on the comparison between the observed levels of differentiation in gene frequencies among subpopulations with those expected under a neutral model of variation [58], with the objective of identifying those markers (outliers) that deviate significantly from the neutral expectation (see, e.g. [56,59,60]). It is generally assumed that local selection is extended over very small chromosomal regions [61,62], and recent studies suggest that genetic differentiation of markers attached to local adaptation genes does not extend beyond about 1–5 kb around the adaptive loci [45,63,64]. In this situation, the probability of finding markers closely associated with selective loci must be really low even in analyses involving thousands of markers. However, regions of increased differentiation (islands of differentiation; [45]) through “divergence hitchhiking” [65], in which strong divergent selection between diverging populations reduces gene exchange, can reach several megabases sequence size [65,66], and markers such as AFLPs can be appropriate to delineate these regions. In fact, analysis combining QTL mapping and detection of selective loci using AFLP markers show that the distance between the outlier markers and the nearest selected loci ranges 10–32 cM [65,67], which would imply physical distances in the order of megabases. In addition, computer simulations investigating the performance of methods in detecting selective loci under divergent selection with markers such as AFLPs shows that, despite the methods having substantial uncertainty, the average distance between detected outlier markers and true selective loci ranges between 7 and 18 cM [68], in agreement with empirical observations.

## Conclusions

*In-silico AFLP* analyses assessing the distribution of AFLP markers across the genomes of nine eukaryotic species indicates that AFLP bands do not particularly accumulate around (peri) centromeric regions. The percentage of AFLP markers positioned within gene sequences is usually larger than that expected by chance because of their higher GC content relative to intergene sequences. In fact, the use of enzyme pairs recognizing restriction sites with a larger GC content substantially increases the above percentages. Thus, enzymes with high GC content recognition sites should be used if the interest is to obtain markers within or close to gene sequences. The high coverage of AFLP markers across the genomes and the high proportion of markers within or close to gene sequences make them suitable for genome scanning and identifying large islands of genomic differentiation. However, their use in the search for markers closely linked to selected loci for specific traits can be a difficult task, as only a small percentage of markers are expected to be close to particular genes of interest.

## Methods

Whole genome sequences and data on gene positions on the genome were obtained from 9 eukaryotic species (*Homo sapiens, Oryza sativa*, *Anopheles gambiae*, *Drosophila melanogaster*, *Arabidopsis thaliana*, *Caenohabditis elegans*, *Plasmodium falciparum*, *Saccharomyces cerevisiae* and *Schizosaccharomyces pombe*) obtained from the NCBI Entrez Genome database. These species were chosen because of their high coverage of genome sequencing, their assignment of all sequences to chromosomal locations, and because they cover a wide spectrum of genome sizes. A computer program written in C [23] was used to simulate the cutting of the whole genome with two restriction enzymes so as to produce AFLP fragments. We mainly considered the typical enzymes used in AFLP studies, *Eco*RI and *Mse*I (cutting at sites GAATTC and TTAA, respectively), but analyses were also carried out with restriction enzymes with a balanced AT/GC recognition sequence (*Bsm*I and *Taq*I, with sites GAATGC and TCGA, respectively) and with a biased GC composition (*Sac*I and *Hpa*II, with sites GAGCTC and CCGG, respectively). Only fragments *Eco*RI-*Mse*I, *Bsm*I-*Taq*I or *Sac*I-*Hpa*II with sizes between 40 and 440 nucleotides (which correspond to PCR fragments between 72 and 472 when the typical primers are added) were used to mimic the experimental procedure used in AFLP studies. The distance in base-pairs between consecutive AFLP fragments and between each AFLP fragment and its closest gene were recorded.

In order to illustrate the number of AFLP markers closest to specific sets of genes, three examples of candidate loci were analysed. These correspond to 46 candidate genes for Aluminium tolerance in *Oryza sativa*[53], 51 candidate genes for flowering time in *Arabidopsis thaliana*[69], and 102 candidate genes for developmental time in *Drosophila melanogaster*[70]. The locations of these candidate genes were searched for in the GENBANK (*Drosophila* and *Arabidopsis*) and PLANTPAN (*Oryza*) databases, but only 42, 50 and 89 genes (respectively) were localised and considered in the analysis.

## Competing interests

The authors declare they have no competing interests.

## Authors’ contributions

All authors contributed to the design of the study and the writing of the manuscript. AC and MJGP carried out the computer analyses. All authors read and approved the final manuscript.

## Authors’ information

The authors are members of the Population Genetics and Cytogenetics Group of the University of Vigo (http://webs.uvigo.es/genxb2/).

## Supplementary Material

Additional file 1 **Distribution of the number of AFLP bands (*Eco*RI/*Mse*I) (in red) and the number of genes (in blue) across the different species, shown in non-overlapping windows of 100 or 200 kb. (S1)***Homo sapiens* (regions with no markers and genes denote unsequenced genomic areas)*.***(S2)***Oryza sativa* (regions with no markers and genes denote unsequenced genomic areas)*.***(S3)***Anopheles gambiae*. **(S4)***Drosophila melanogaster.***(S5)***Caenorhabditis elegans.***(S6)***Plasmodium falciparum.***(S7)***Schizosaccharomyces pombe.***(S8)***Saccharomyces cerevisiae.* Click here for file
