# Supplementary material for: Genomic distribution of AFLP markers relative to gene locations for different eukaryotic species
Source: BMC Genomics. 2013 Aug 1;14:528. doi: 10.1186/1471-2164-14-528 (PMC3750350; doi:10.1186/1471-2164-14-528)
Supplement: Additional file 1 — Distribution of the number of AFLP bands (EcoRI/MseI) (in red) and the number of genes (in blue) across the different species, shown in non-overlapping windows of 100 or 200 kb. (S1)Homo sapiens (regions with no markers and genes denote unsequenced genomic areas).(S2)Oryza sativa (regions with no markers and genes denote unsequenced genomic areas).(S3)Anopheles gambiae. (S4)Drosophila melanogaster.(S5)Caenorhabditis elegans.(S6)Plasmodium falciparum.(S7)Schizosaccharomyces pombe.(S8)Saccharomyces cerevisiae. [file 1471-2164-14-528-S1.doc]

**Figure S1**

**
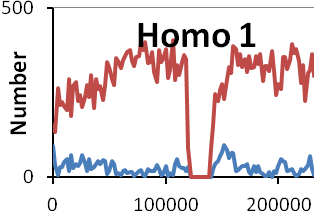

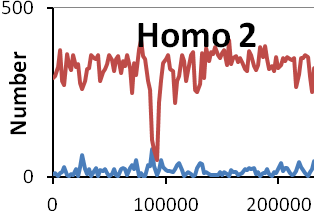

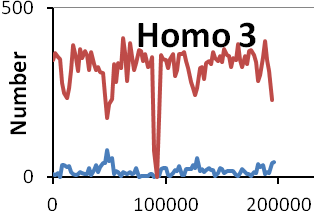

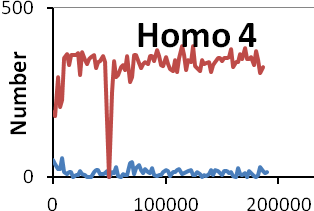

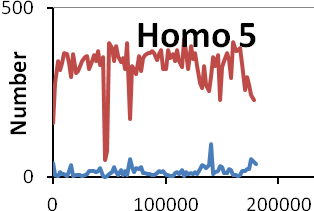

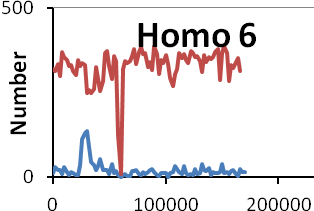

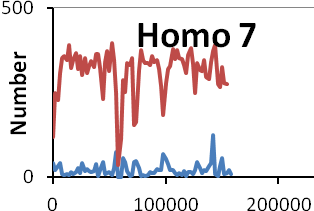

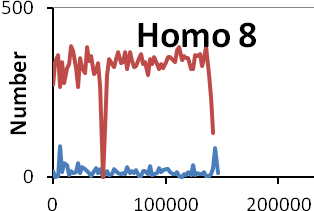

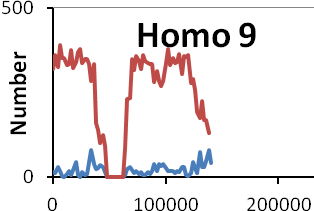

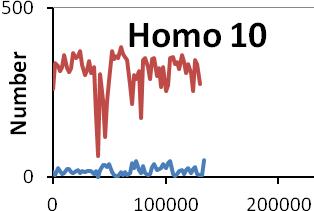
**

**
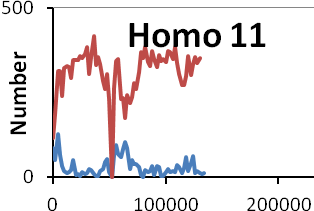

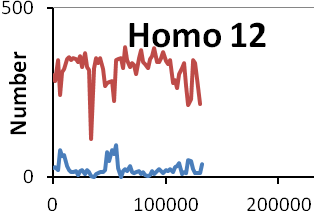
**

**Figure S1 (cont.)**

**
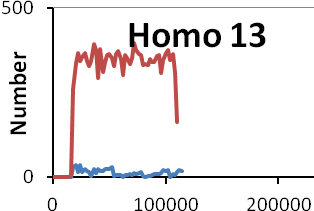

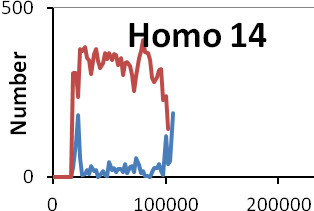

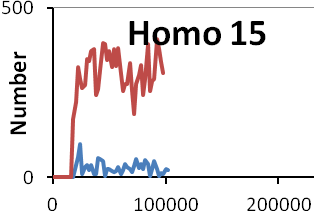

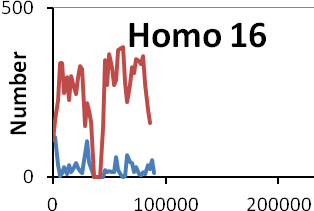

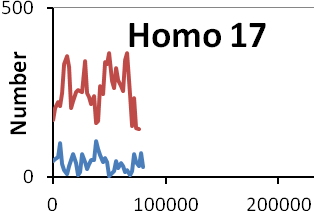

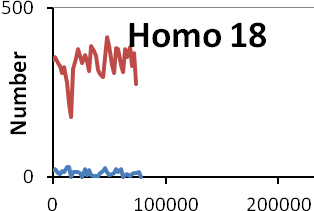

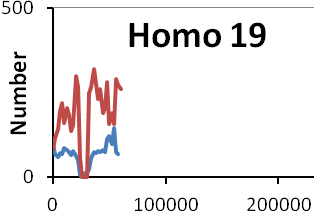

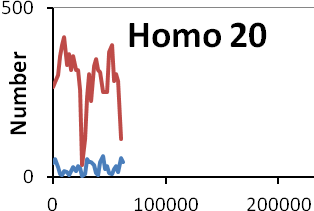

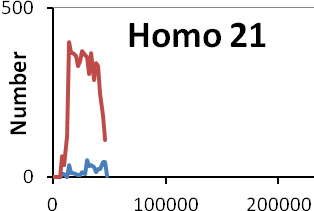

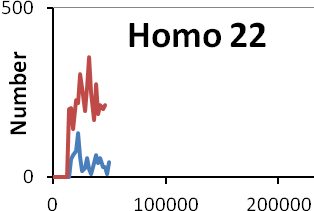

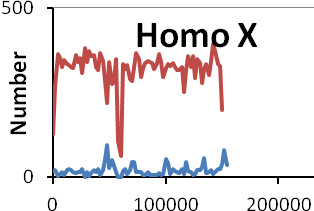

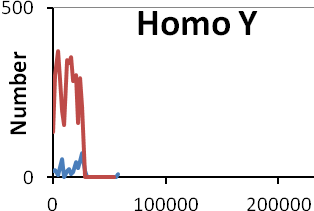
**

**Figure S2**

**
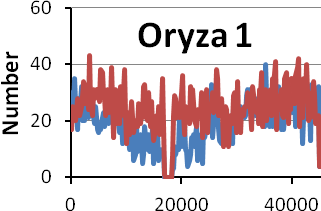

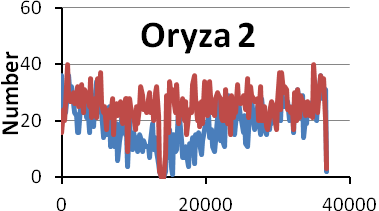

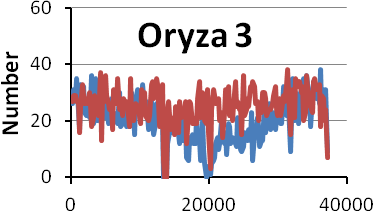

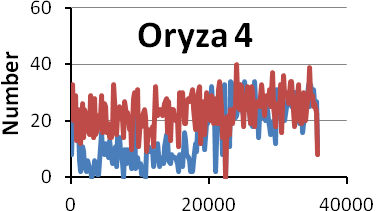

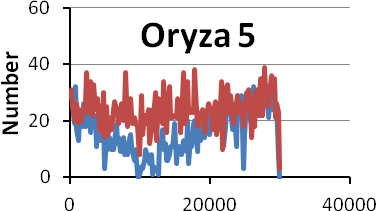

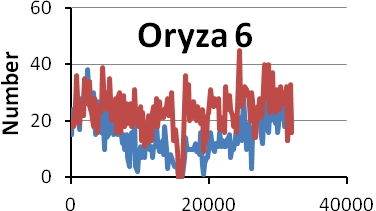

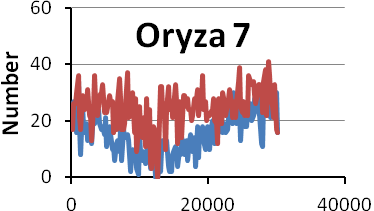

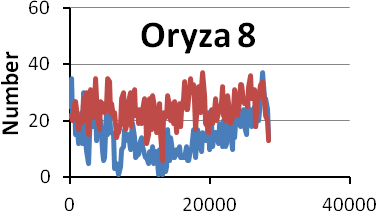

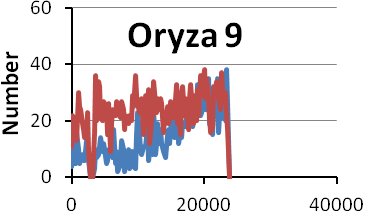

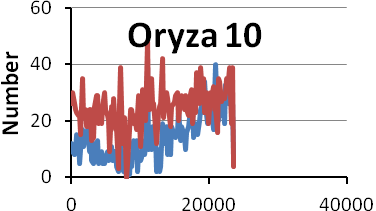

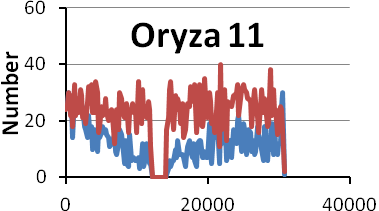

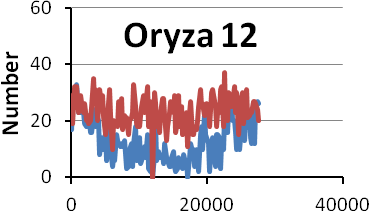
**

**Figure S3**

**
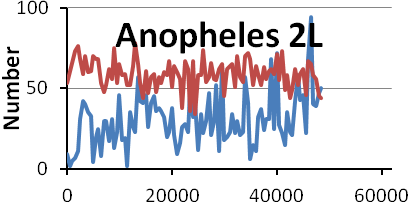

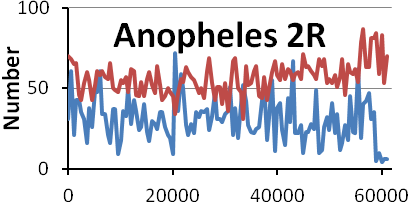

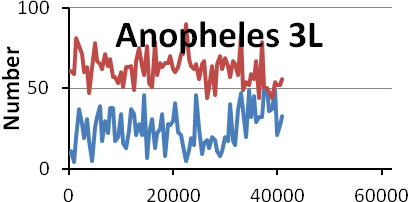

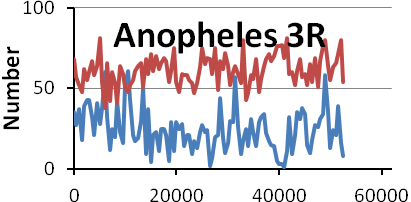

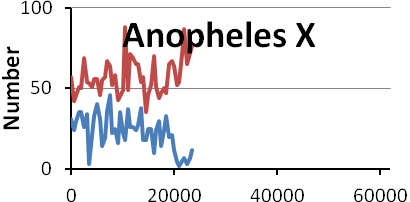
**

**Figure S4**

**
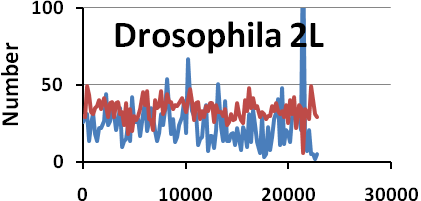

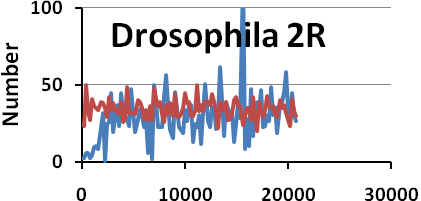

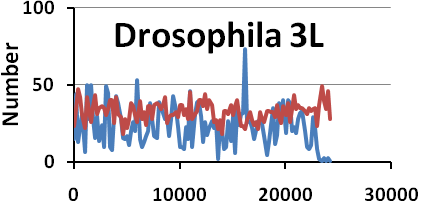

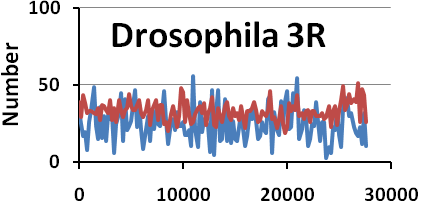

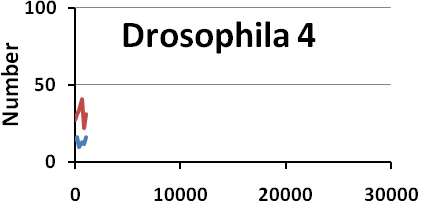

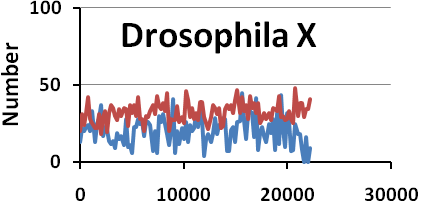
**

**Figure S5**

**
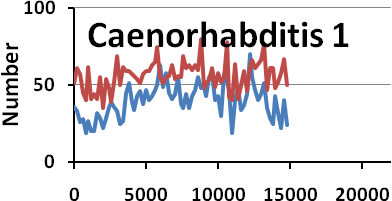

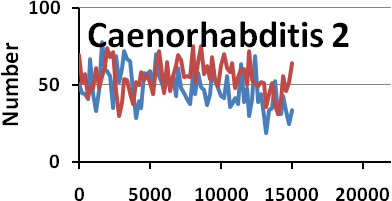

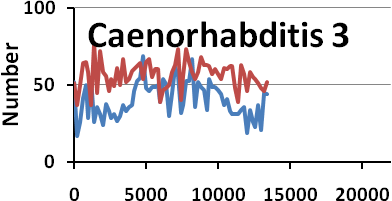

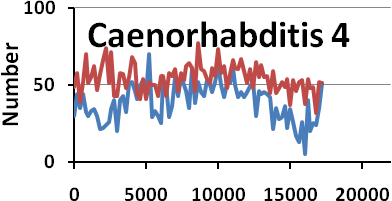

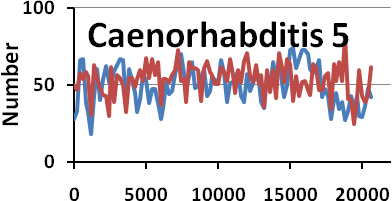

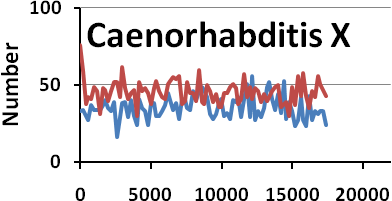
**

**Figure S6**

**
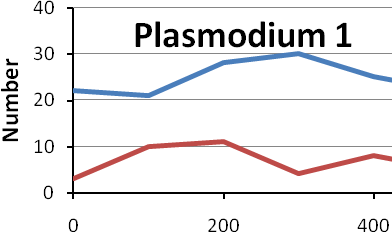

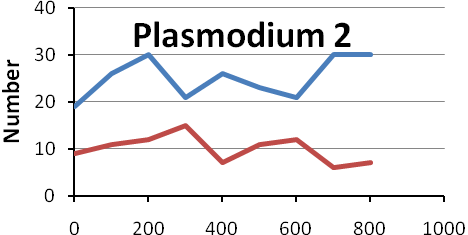

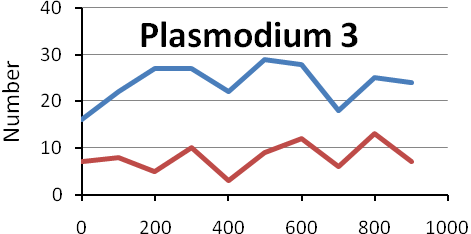

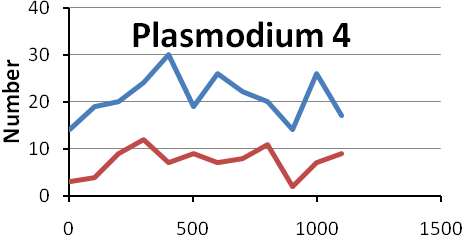
** **
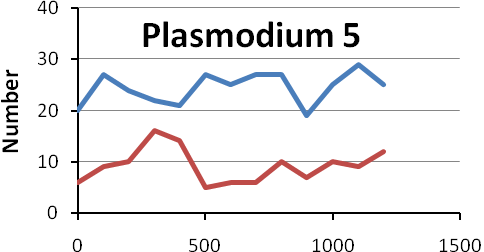

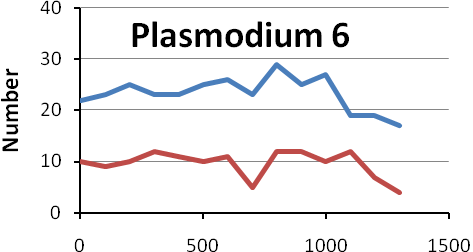
**

**
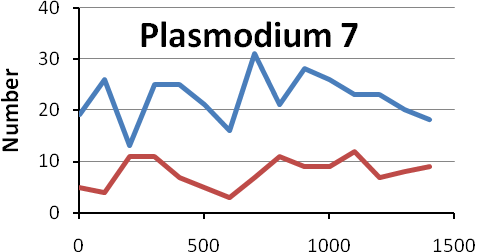

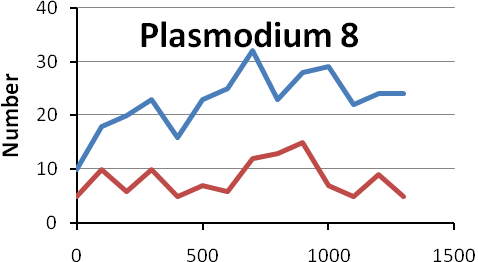
**

**Figure S6 (cont.)**

**
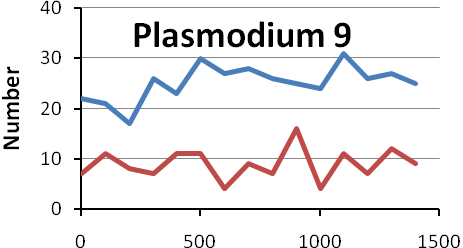

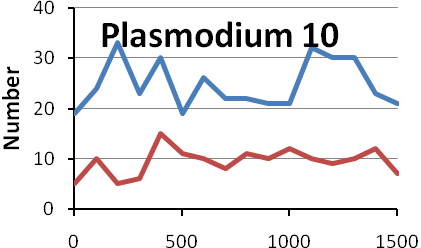

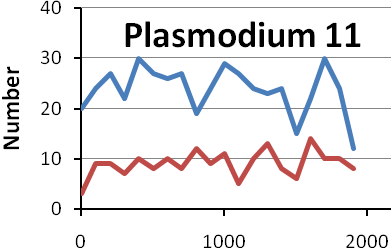

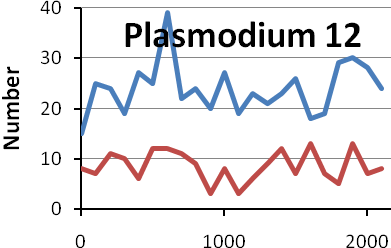

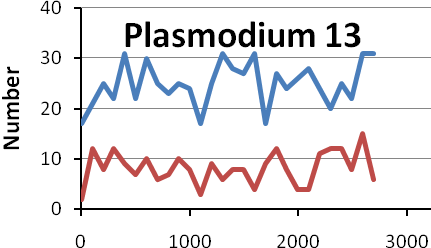

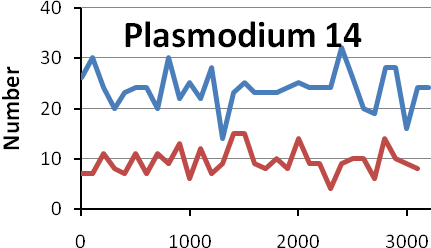
**

**Figure S7**

**
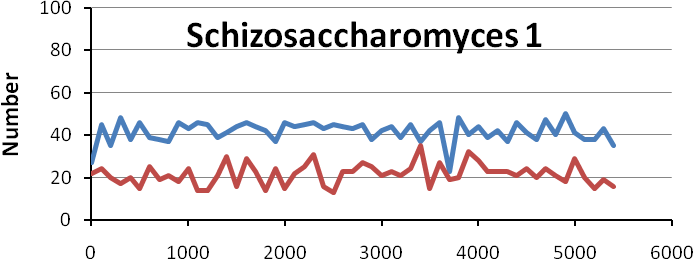

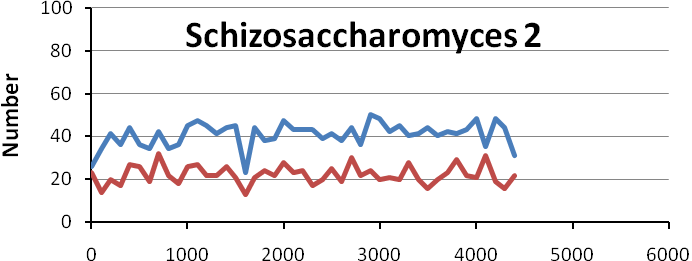

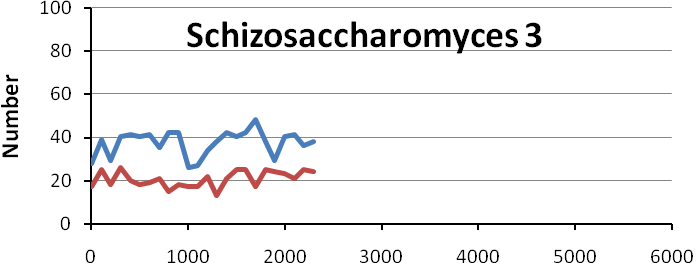
**

**Figure S8**

**
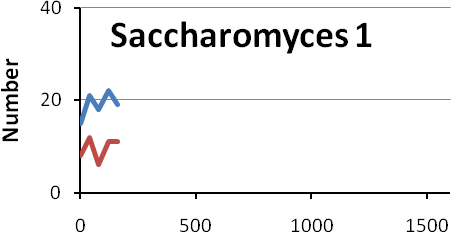

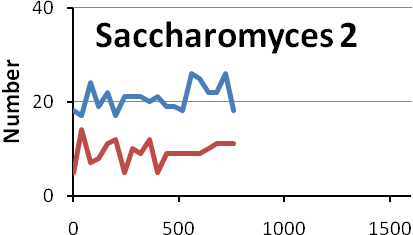

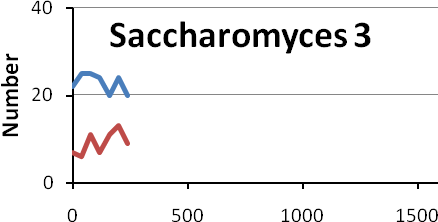

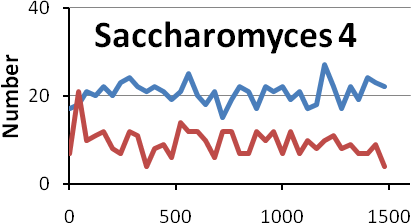

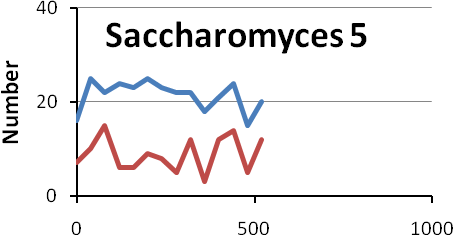

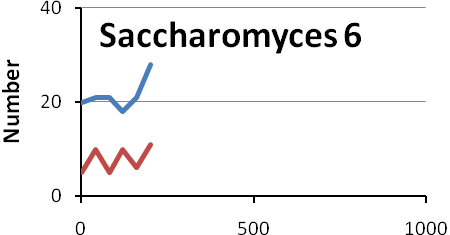
**

**
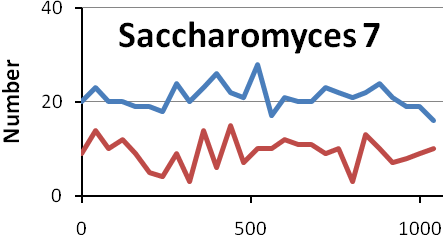

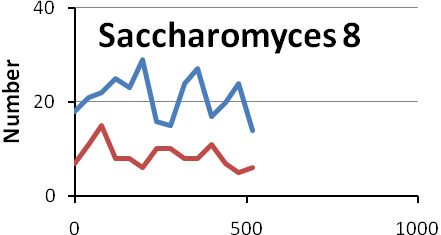
**

**Figure S8 (cont.)**

**
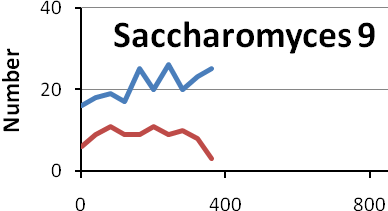

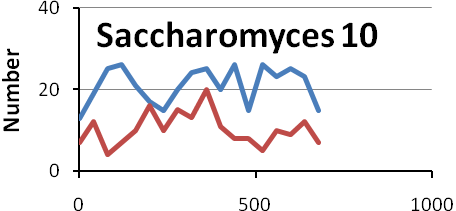

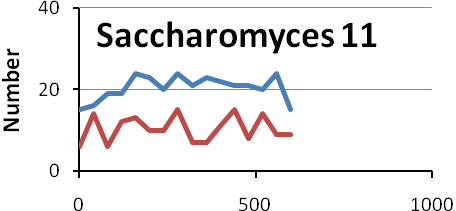

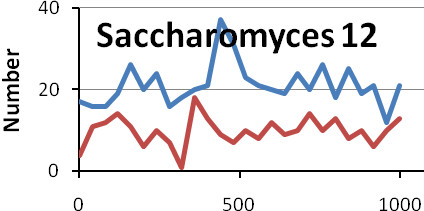

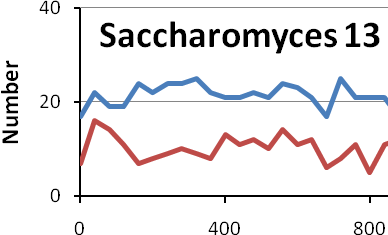

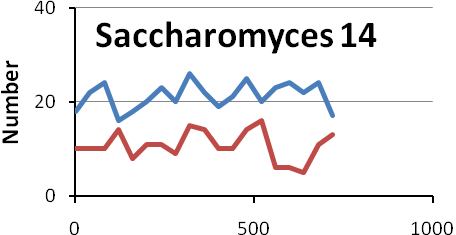

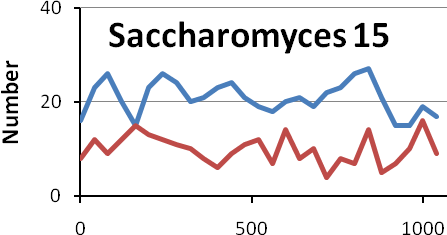

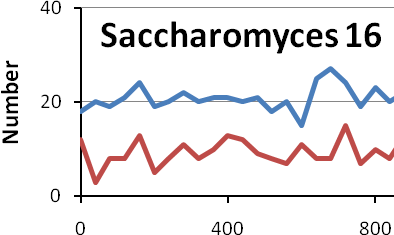
**
